# Biomechanical properties of lumbar vertebral ring apophysis cage under endplate injury: a finite element analysis

**DOI:** 10.1186/s12891-023-06792-1

**Published:** 2023-08-30

**Authors:** Jian Wang, Ziming Geng, Jincheng Wu, Jianchao Liu, Zepei Zhang, Jun Miao, Ruihua Li

**Affiliations:** 1 Department of Spine Surgery, Tianjin Hospital, Tianjin University, No. 406 Jiefang South Rd, Hexi District, Tianjin, 300211 China; 2 The Second Affiliated Hospital of Hainan Medical University, Haikou City, Hainan, China

**Keywords:** Lateral Lumbar Interbody Fusion (LLIF), Biomechanical evaluation, Finite element analysis, Cage subsidence

## Abstract

**Objective:**

This study aimed to compare the biomechanical properties of lumbar interbody fusion involving two types of cages. The study evaluated the effectiveness of the cage spanning the ring apophysis, regardless of the endplate's integrity.

**Methods:**

A finite element model of the normal spine was established and validated in this study. The validated model was then utilized to simulate Lateral Lumbar Interbody Fusion (LLIF) with posterior pedicle screw fixation without posterior osteotomy. Two models of interbody fusion cage were placed at the L4/5 level, and the destruction of the bony endplate caused by curetting the cartilaginous endplate during surgery was simulated. Four models were established, including Model 1 with an intact endplate and long cage spanning the ring apophysis, Model 2 with endplate decortication and long cage spanning the ring apophysis, Model 3 with an intact endplate and short cage, and Model 4 with endplate decortication and short cage. Analyzed were the ROM of the fixed and adjacent segments, screw rod system stress, interface stress between cage and L5 endplate, trabecular bone stress on the upper surface of L5, and intervertebral disc pressure (IDP) of adjacent segments.

**Results:**

There were no significant differences in ROM and IDP between adjacent segments in each postoperative model. In the short cage model, the range of motion (ROM), contact pressure between the cage and endplate, stress in L5 cancellous bone, and stress in the screw-rod system all exhibited an increase ranging from 0.4% to 79.9%, 252.9% to 526.9%, 27.3% to 133.3%, and 11.4% to 107%, respectively. This trend was further amplified when the endplate was damaged, resulting in a maximum increase of 88.6%, 676.1%, 516.6%, and 109.3%, respectively. Regardless of the integrity of the endplate, the long cage provided greater support strength compared to the short cage.

**Conclusions:**

Caution should be exercised during endplate preparation and cage placement to maintain the endplate's integrity. Based on preoperative X-ray evaluation, the selection of a cage that exceeds the width of the pedicle by at least 5 mm (ensuring complete coverage of the vertebral ring) has demonstrated remarkable biomechanical performance in lateral lumbar interbody fusion procedures. By opting for such a cage, we expect a reduced occurrence of complications, including cage subsidence, internal fixation system failure, and rod fracture.

## Introduction

Lateral lumbar interbody fusion (LLIF) is a minimally invasive surgical technique used to treat lumbar degenerative diseases. This technique provides direct access to the intervertebral space via lateral transpsoas surgical access, allowing surgeons to deploy an interbody fusion cage that spans the entire vertebral endplate. This unique approach enables the correction of deformities and nerve decompression in a minimally invasive manner [1]. "This surgical procedure can result in better recovery, improved pain, and functional outcomes. The ultimate goal of the surgery should not involve any compromise to reduce complications. By preserving ligamentous structures and inserting larger intervertebral implants, disc height and stability can be restored, which may indirectly improve foraminal volume and lead to a reduction in radiculopathy [2].

During LLIF surgery, to ensure postoperative spinal stability and good outcomes, pedicle screw fixation of the corresponding segment is generally performed in addition to the standalone cage, which can lead to many complications [3–6]. Despite rigid fixation using a screw-rod system, cage subsidence during lateral fusion remains a concern for surgeons because it undermines the goals of interbody fusion surgery, such as restoration of disc or foraminal height, indirect decompression, and segmental alignment. Currently, the predictors of subsidence mainly include the use of a small interbody fusion cage, a low bone mineral density score, and aggressive endplate preparation [6–8]. The vertebral endplates refer to the thin cortical bone that exists on the upper and lower surfaces of the vertebral body. Histological studies by Hou et al. have shown that the endplate is not purely cortical bone, but instead, a porous structure with trabecular involvement [9]. The significance of the endplate has been confirmed in numerous studies, with reports indicating that its removal significantly reduces the structural properties of the lumbar spine [9, 10]. Recent studies have shown that although there is no obvious destruction of the endplate before cage placement, the endplate can collapse due to the cage's extrusion during placement, leading to subsidence in a short period after the operation [11]. Compared to the weaker central portion supported by cancellous bone, the ring apophysis has been found to be the strongest part of the vertebral endplate, consisting of a cortical bone margin surrounding it [12, 13]. When selecting the type of cage, it is recommended to choose a longer cage that can be placed on both sides of the epiphyseal ring to provide maximum support [2]. The efficacy of cages with various shapes and sizes in fusion surgery has been previously investigated [1, 6], however, no biomechanical analysis has been conducted to evaluate the performance of the same type of cage under endplate destruction.

The validated finite element model permits the adjustment of both the geometric shape and various material properties to meet design requirements. As a result, for a particular study,it can more accurately reflect the interactions between various structures. Meanwhile, the experimental conditions can be directly compared, which helps to avoid the influence of individual differences in experimental materials on the results and enhance the accuracy of the analysis [14]. Taking into account the factor of endplate integrity during the operation, the experiment utilized the finite element simulation method to establish two types of cages, one with normal endplate and the other with the same degree of endplate damage, for biomechanical analysis. The long cage is a cage that spans the ring apophysis, while the short cage only covers the upper part of the endplate. We hypothesize that the long cage spanning the vertebral ring apophysis has better biomechanical properties than the short cage, and that it can still maintain strong anti-settlement ability even in the case of endplate failure.

## Materials and methods

### Normal finite element model

The data for the L1-S lumbar spine model were obtained from a healthy adult male volunteer (28 years old, weight 72kg, height 173cm) with no history of spinal trauma or spinal diseases, as confirmed by clinical imaging examinations, in order to establish a normal finite element model. The volunteer was recruited from the Department of Spine Surgery at Tianjin Hospital. Informed consent was obtained in accordance with relevant regulations and submitted to the Ethics Committee of Tianjin Hospital for approval. All research procedures were strictly conducted in accordance with the principles of the Declaration of Helsinki. Thin-slice 64-slice spiral CT (Siemens, Erlangen, Germany) was used to obtain tomographic data in DICOM format with a slice spacing of 0.625mm, which included imaging data of one sacrum, five vertebral bodies, and intervertebral discs. The model reconstruction method was consistent with the previous experimental study [15]; the image data were imported into mimics20.0 (Materials, Leuven, Belgium), and the 3D geometric surface model of the lumbar spine was reconstructed and saved in STL format [16]. The 3D geometric model of the lumbar spine was processed using 3-Matic 12.0 software (Materialise Inc.), which included functions such as wrapping, smoothing, and Boolean operation. The redundant triangular surfaces were removed to generate more detailed 3D images, and initial structures of facet joints, intervertebral discs, and nucleus pulposus were constructed [17]. The 3D surface models of the lumbar spine were processed using smoothing and accurate surface features in Geomagic Studio 12.0 (Geomagic, North Carolina). The processed models were then imported into Hypermesh2017 (Altair, Troy, Michigan, USA), where meshing was performed and seven ligaments (anterior longitudinal ligament (ALL), posterior longitudinal ligament (PLL), transverse ligament (ITL), capsular ligament (CL), ligamentum flavum (LF), interspinous ligament (ISL), and supraspinous ligament (SSL)) were constructed. Finally, Abaqus 2019 (Simulia, Johnston, RI, USA) was used for model assembly, attribute assignment, and finite element analysis [18, 19].

**Fig. 1:**
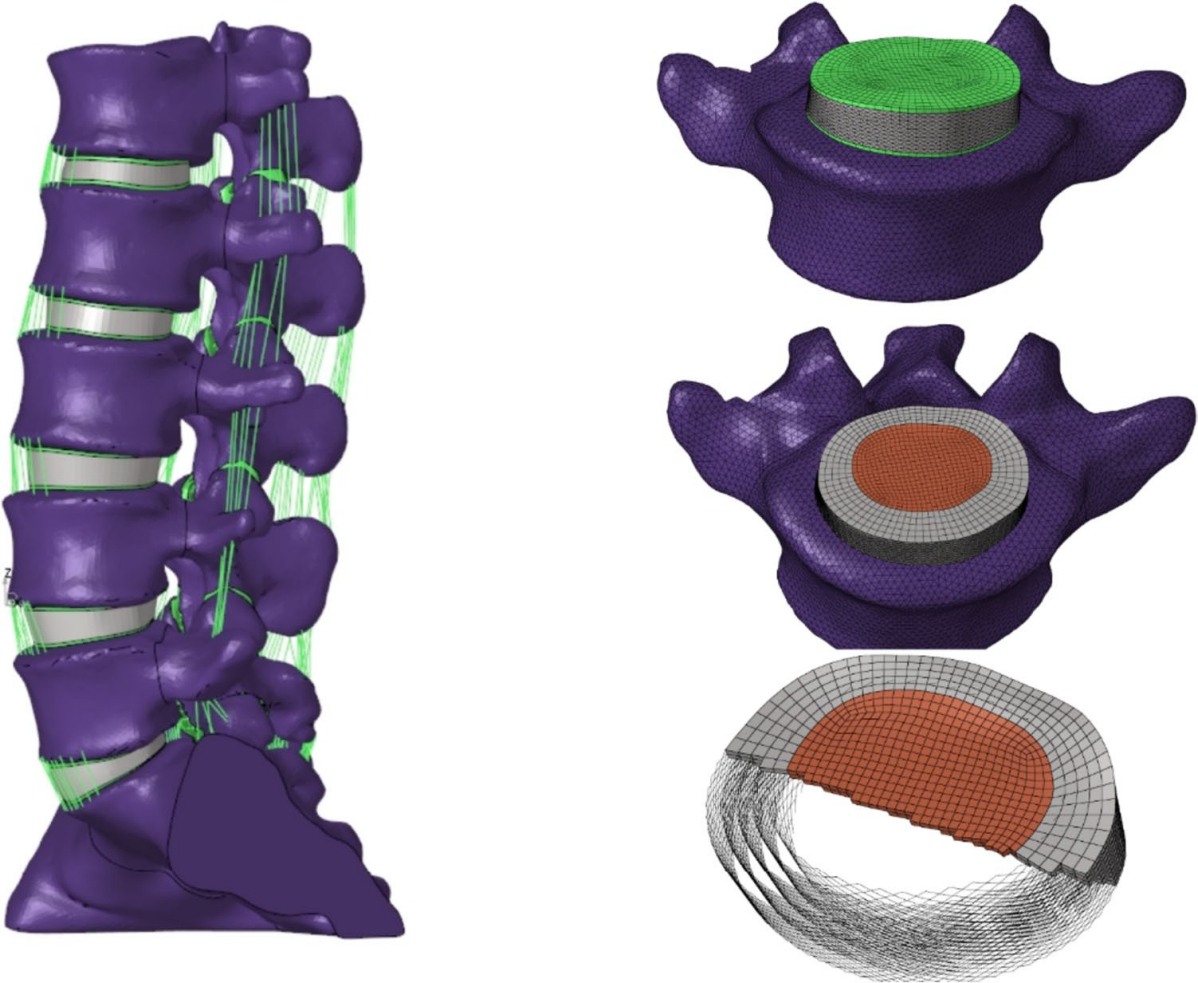
Finite element models of the intact lumbar spine and intervertebral disc structures

In this study, we reconstructed a 3D finite element model of the normal L1-S lumbar spine, as shown in Fig. 1. The intervertebral disc was represented by a hexahedral mesh that included the annulus fibrosus matrix, nucleus pulposus, annulus fibrosus fibers, and endplate. Both the upper and lower endplates were modeled to be 0.5mm thick [20], The nucleus pulposus accounts for 30 to 40% of the disc area [21–23]. The thickness of cortical bone and articular cartilage were 1 mm and 0.2 mm, respectively [16, 20]. The truss element, which only bears tensile forces, was utilized to simulate the ligament and annulus fibrosus. The annulus fibrosus was constructed in five layers from the inside out and was embedded in the annulus fibrosus matrix at a ± 30° tilt angle. The elastic strength increased proportionally from 360 MPa in the innermost layer to 550 MPa in the outermost layer [21, 24]. The full L1-S model contains 1011182 units and 248371 nodes, defined using material properties according to previously reported literature (Table 1) [20, 21, 25, 26].

**Table 1:** Material properties used by finite element model

Component	Young’s Modulus (MPa)	Poisson Ratio	Cross-Sectional Area(mm2)
Vertebra			
Cortical bone	12,000	0.3	
Cancellous bone	100	0.2	
Posterior element	3500	0.25	
Sacrum	5000	0.2	
Facet	11	0.2	
Disc			
Endplate	24	0.4	
Nucleus pulpous	1	0.49	
Annulus ground substance	2	0.45	
Annulus fibers	360–550		0.15
Ligaments			
ALL	7.8		63.7
PLL	10		20
LF	15		40
CL	7.5		30
ISL	10		40
SSL	8		30
ITL	10		1.8
Implants			
Cage (titanium alloy)	110,000	0.3	
Bone graft	100	0.2	
Screws and rods (titanium alloy)	110,000	0.3	

### Model simulation

Lumbar disc herniation is one of the most common spinal pathologies, often occurring at the L4/L5 and L5/S1 levels [27]. We selected the L4/5 segment for lateral lumbar interbody fusion (LLIF) in this study. Based on the characteristics of clinical LLIF surgery, we completely removed the L4-5 annulus fibrosus and nucleus pulposus, and placed two pedicle screws in L4 and L5 according to Weinstein's standard [28], which were then fixed with titanium rods. For this experiment, the internal fixation instruments necessary for the simulated surgery were designed using Pro/Engineer 5.0 software. To obtain a convenient internal fixation model for analysis, a simplified lumbar pedicle screw (diameter 6.5mm, length 45mm) and connecting rod (diameter 5.5mm) were designed, taking into consideration the surgical methods used in previous studies.

The Pro/Engineer software was utilized to design two types of cages: a long cage, which was 5mm longer than the outer pedicular width to ensure that it spanned the ring apophysis on each side of the specimen in Model 1 and 2, and a short cage for Model 3 and 4. The short cage was about the same size as the endplate, covering just above the endplate, and made of titanium alloy [29]. The dimensions of the two cages in this study were based on the actual situation of the model, as well as previous studies, with the long cage measuring 56*18*12mm and the short cage measuring30*18*12mm [30, 31].

**Fig. 2:**
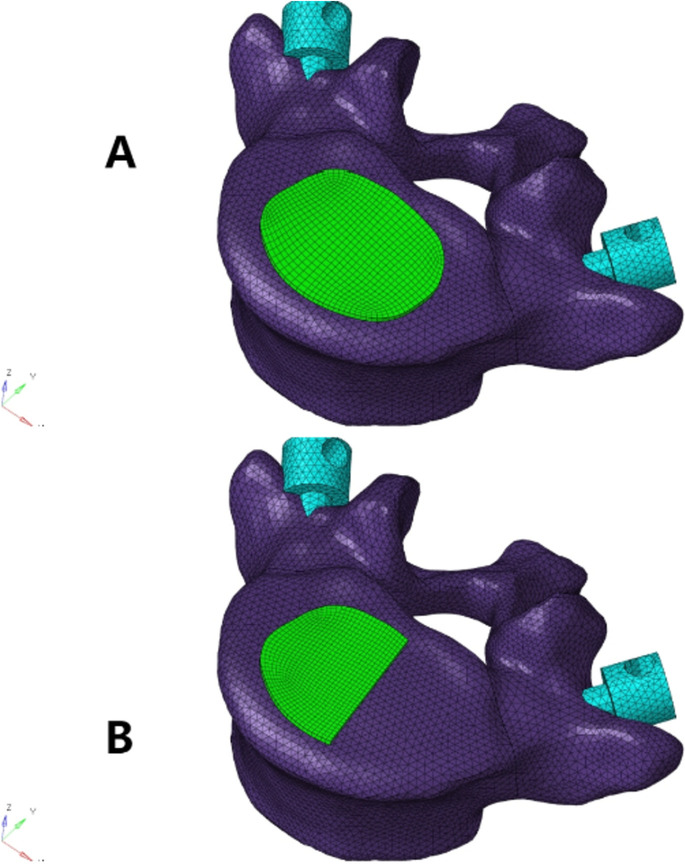
The endplate failure model was simulated. **A** Complete endplate. **B** Partial destruction of the left endplate

**Fig. 3:**
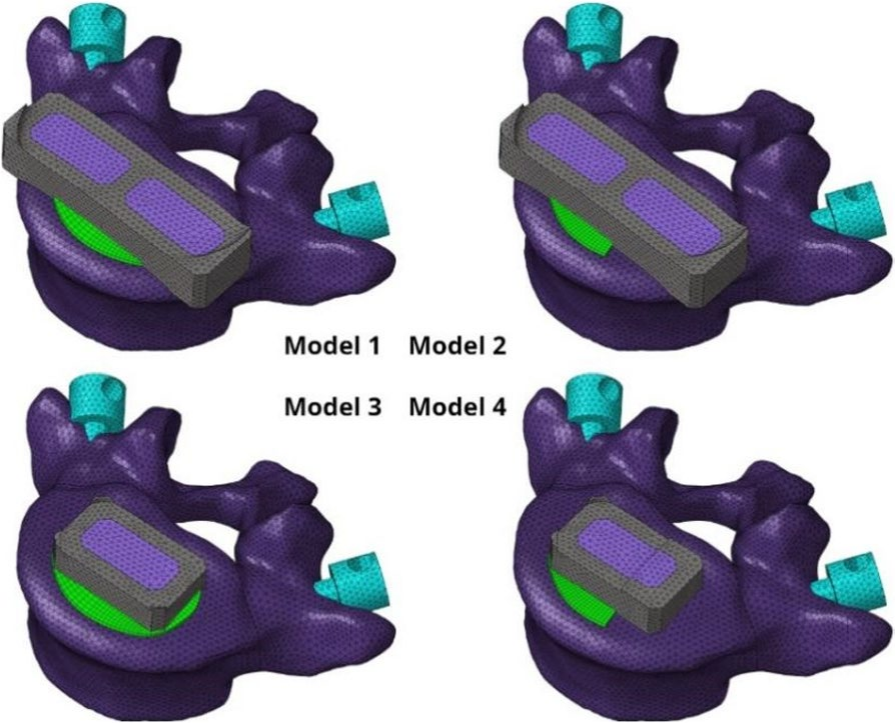
Four models were finally established. Model 1: intact endplate with long cage spanning the ring apophysis; Model 2: endplate decortication with long cage spanning the ring apophysis; Model 3: Intact endplate with short cage; Model4: endplate decortication with short cage

According to the opinions of multiple experienced surgeons, the upper surface of the L5 vertebral body in the established model was processed, and the left side of the L5 superior endplate was removed to simulate the situation of iatrogenic bony endplate destruction caused by the removal of the cartilage endplate (refer to Fig. 2). The cage was placed in the center of the intervertebral space, and binding constraints were used at the junction between the screw-rod system, cage, and the model involved in the simulation surgery to form a rigid connection that restricted the movement of the vertebral body, screws, cage, and bone graft [32, 33]. To simulate graft fusion under internal fixation, the cancellous bone was filled in all caged graft holes. A Boolean operation was performed to remove the part in contact with the vertebral body, ensuring geometric contact between the vertebral body and the graft. Four models were established, as shown in Fig. 3. It should be noted that screw sliding in the vertebral body was not considered in this study. To simplify the analysis, the screw thread was removed without affecting the results of the study [34, 35].

### FE model validation

In this experiment, we verified the rationality of the experimental model by referring to the research method previously used by Renner et al. [36]. In accordance with previous research, we constrained the base of the sacrum to limit its displacement and rotation in all directions. Spinal motion was defined in the sagittal, coronal, and transverse planes as flexion and extension, lateral bending, and rotation, respectively. Four pure bending moments (8 N·m in flexion, 6 N·m in extension, ± 6 N·m in lateral bending, and ± 4 N·m in rotation) were applied to the center of the upper surface of L1 to simulate lumbar spine motion. The ROM for each segment was measured and compared to the results of previous studies. Aside from confirming the range of motion (ROM) for each lumbar segment, we also validated the intervertebral disc pressure (IDP) specifically in the L4/5 segment. Drawing upon prior research conducted by Brinckmann et al. [37], we measured the IDP of the L4/5 segment by incrementally applying compressive forces (300N, 1000N).

### Boundary and loading conditions

We utilized ABAQUS software for the analysis and calculation of the final model. Firstly, we imported each model component in INP format to assemble the model. Then, we constrained the boundaries of the model and applied load simultaneously. An axial load of 280N was applied to the L1 vertebral body to simulate the weight borne by the lumbar spine in the human body [20, 38]. The lumbar spine was subjected to a bending moment of 7.5 N ·m to simulate flexion, extension, lateral bending, and rotation [15].

### Assessment indexes

In this study, we compared the biomechanical differences between different models by calculating and measuring the range of motion (ROM) of the fixed segment and the adjacent segment in six directions (flexion, extension, lateral bending, and rotation), VMS stress of the screw-rod system, VMS stress of the endplate on the contact surface of the Cage and L5 vertebral body, and the pressure in the nucleus pulposus (IDP) of the adjacent segment. Our objective was to analyze the biomechanical analysis of two different cages under the condition of intact and damaged endplate, and provide some clinical guidance.

## Results

### FE model validation

**Fig. 4:**
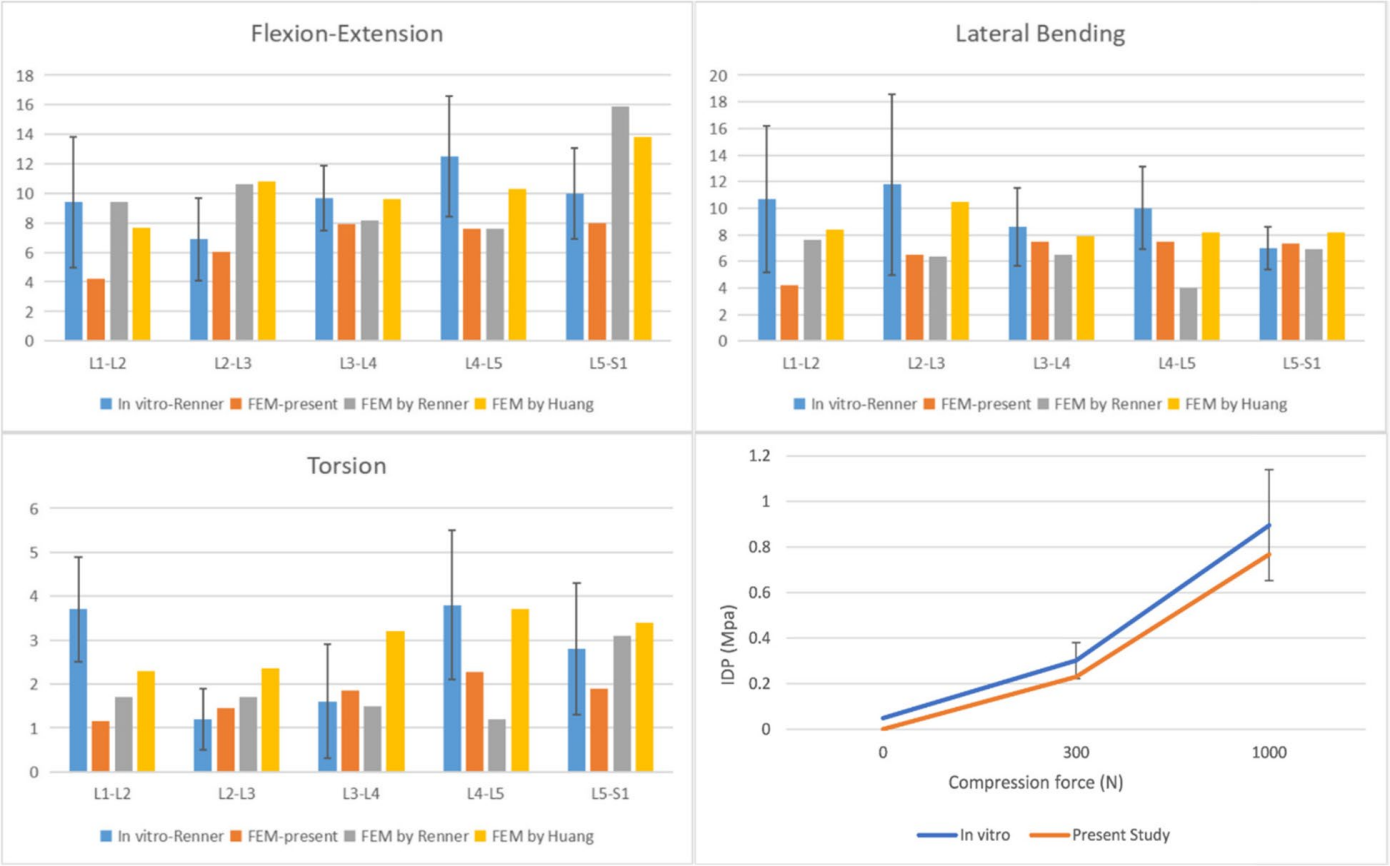
Comparison of the ROM of each motion segment and the IDP of L4/5 between the present and previous studies

The FE model was verified for its rationality using experimental methods that were reported previously. The ROM of L1-S1 and the IDP of L4/5 were measured and compared with the results of previous studies under the same loading and boundary conditions [26, 36, 37], as shown in the Fig. 4. Our findings indicated that the range of motion (ROM) for each segment was consistent with previous studies, with the exception of the L1-2 segment, which was not included in our experimental design. The ROM values for the remaining segments fell within one standard deviation of the referenced studies. And the observed trend of increasing IDP in the L4/5 segment aligns with the findings from previous in vitro experiments conducted under progressively escalating compression loads. Thus, we consider the finite element model used in this study to be valid for subsequent analyses.

### The ROM of the fixed segment

**Fig. 5:**
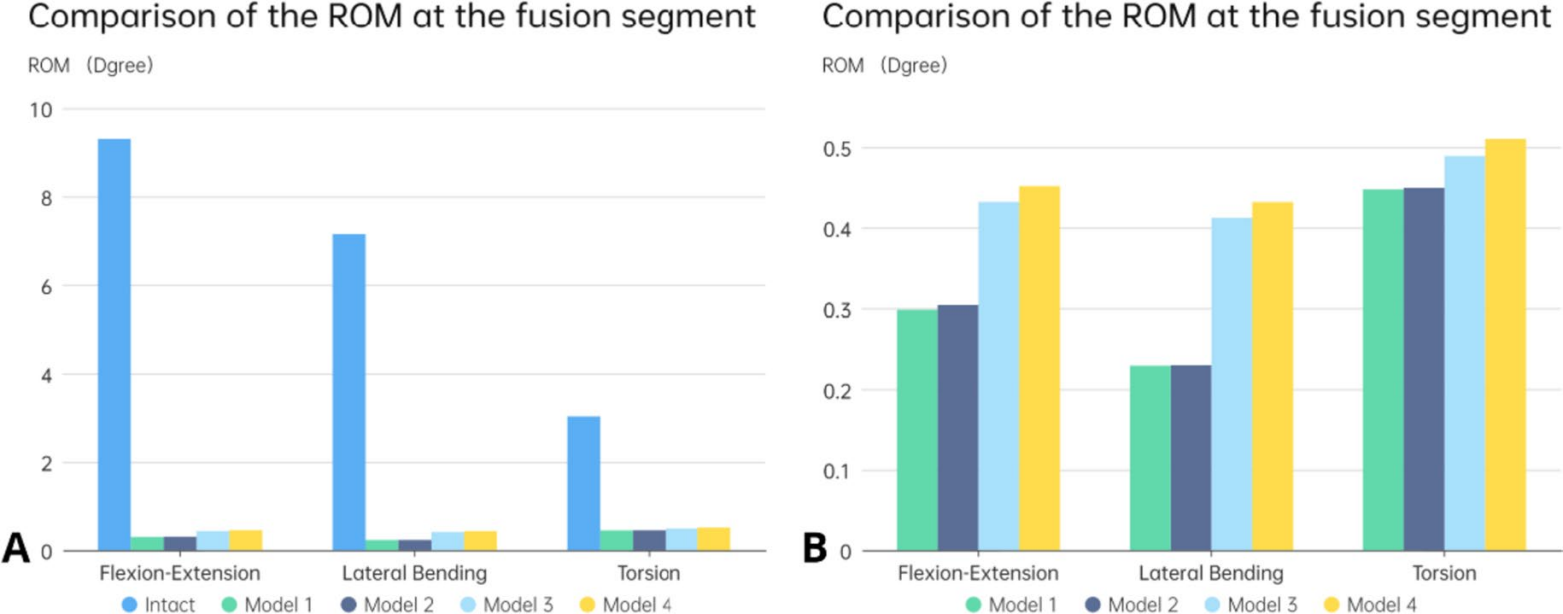
Comparison of the ROM at the fusion segment. **A** Between the intact and surgical FE models; **B** Comparison between two different cages

Figure 5 illustrates the range of motion of each surgical model and the complete model in the fused segment. Both cages limited the motion of the model in the coronal plane the most, followed by the motion of the model in the sagittal plane, while the vertebral rotation was less restricted. For the long cage, regardless of the integrity of the endplate, it limited the motion of the coronal plane the most (96.8%) compared to the normal model, and the motion of the vertebral body in the transverse plane the least (85.5%). However, the endplate's condition did not significantly affect the mobility of the long cage during the fixation stage. In contrast, the range of motion of the short cage was higher than that of the long cage in all directions, with the most significant difference observed in the left and right lateral bending of the vertebral body, followed by the flexion and extension movement, and the smallest difference was observed in the rotation movement of the vertebral body (48.8%, 33.3%, 13.7% more than the long cage, respectively). Furthermore, under the condition of endplate destruction, the ROM of the short cage increased significantly in several directions (the average ROM increased by about 5%). In general, the long cage had the smallest range of motion on all sides, regardless of whether the endplate was damaged or not. On the other hand, the range of motion of the model using the short cage increased. If the endplate is damaged, the range of motion limiting ability of the short cage will decrease by about 5%.

### The intradiscal pressure (IDP) and range of motion (ROM) at L3-4 in the normal model and fusion models

**Fig. 6:**
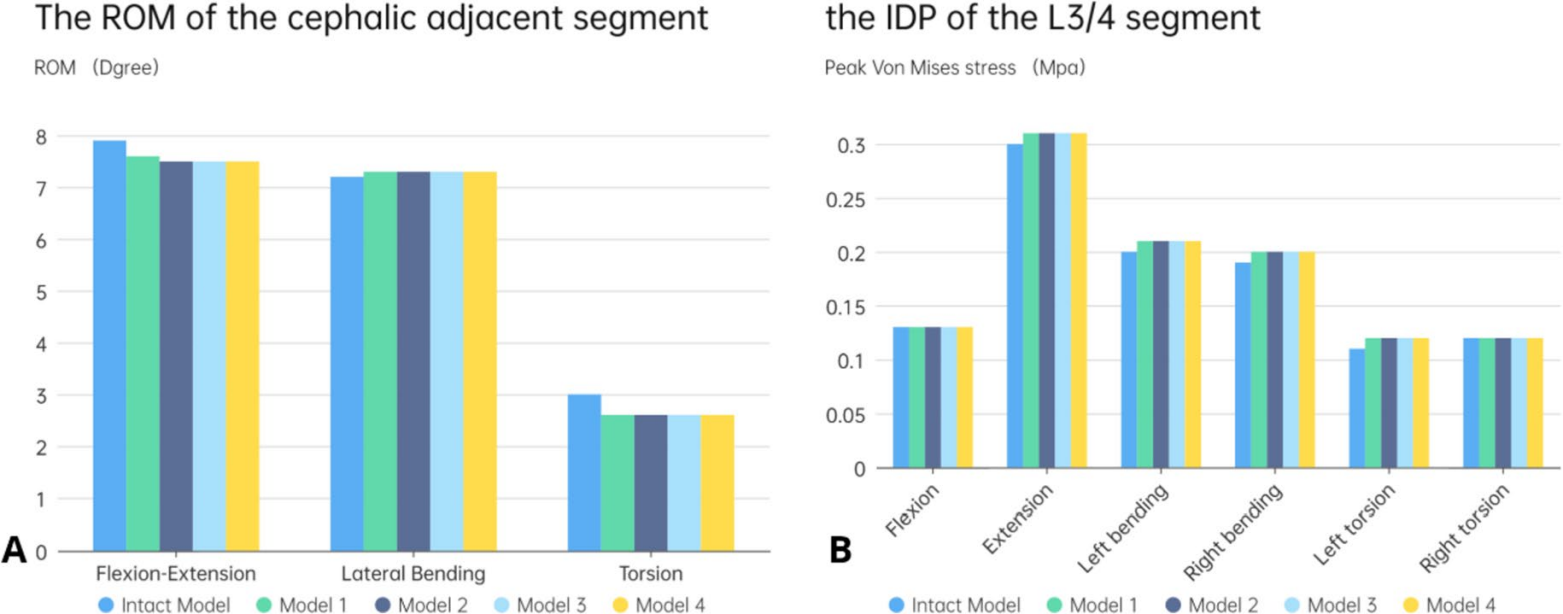
The intradiscal pressure (IDP) and range of motion (ROM) at L3-L4. A:the ROM of L3-L4; B:the IDP of L3-L4

The range of motion of each model in the adjacent segment (L3/4) is displayed in Fig. 6A. The range of motion of the L3-4 segment in the complete model was less than that in the surgical simulation model only in lateral bending. Conversely, the range of motion of the postoperative model was reduced in flexion, extension, and rotation movements, but the maximum difference was only 0.4° in rotation movement. Regardless of the variation in the cage or endplate integrity, there was little alteration in the range of motion of the adjacent segment in the model after simulated surgery.

The intradiscal pressures in the adjacent segments of the intact model and each surgical model are presented in Fig. 6B. The results indicate that the pressure of the intervertebral disc in the adjacent segments of all surgical models was greater than or equal to that of the intact model after fusion fixation of L4/5 segments. However, the difference was not significant overall. Moreover, there was no difference observed in the comparison of flexion and right rotation in several models. Generally, the L3/4 IDP of the surgical model was highest in the sagittal plane, followed by the coronal plane, and the lowest in the transverse plane. Furthermore, there was no significant difference observed in the L3/4 IDP between long cage and short cage with or without endplate destruction.

### The stress of the screw-rod system

**Fig. 7:**
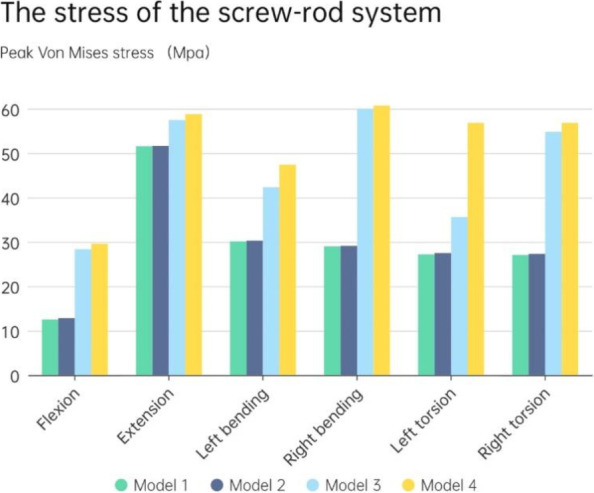
The stress of the screw-rod system

The stress distribution of the internal fixation system provides us with a basis for evaluating postoperative complications such as screw rod fracture and loosening. As illustrated in Fig. 7, the results indicate that in the surgical model with the long cage, the maximum stress of the screw rod system occurred during retroextension (51.6 Mpa), while in the short cage surgical model, the maximum stress appeared during right bending movement (60.7 Mpa). Overall, the total stress in all directions using the long cage was lower than that using the short cage. Additionally, if the endplate was damaged, the force on the model screw rod system using the long cage had little effect, but the force on the model screw rod system using the short cage showed an upward trend. This upward trend was most pronounced during left rotation movement, and the stress increased by approximately 37.3%.

### Stress of the cage‑endplate interface and cancellous bone stress on the upper surface of L5

**Fig. 8:**
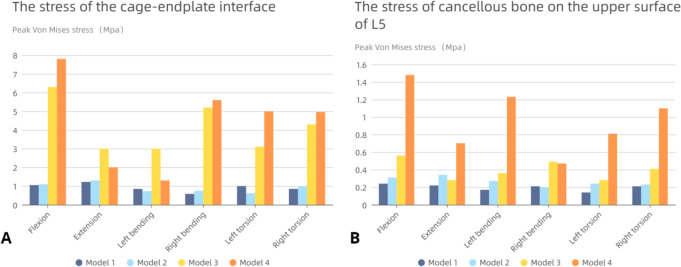
**A** L5 upper surface endplate stress; **B** The stress of cancellous bone on the upper surface of L5

The stresses on the endplates of the four models are presented in Fig. 8A. The stress on the upper L5 endplate of Model 1 and 2 with a Long cage was significantly lower. The minimum stress was observed during right-leaning movement (only 0.58 Mpa), and the maximum stress appeared during backward movement of Model 2 (1.3 Mpa). The endplate stress of Model 2 was smaller than that of Model 1 during left rotation and left bending, respectively. For the model with a short cage, the overall stress was significantly higher than that of Model 1 and 2. The highest stress was observed in Model 4 (7.8 Mpa), and the lowest stress was observed during left bending movement of Model 4 (1.3 Mpa). In general, the Long cage can reduce the stress on the L5 upper endplate, while the short cage has a significant tendency to increase its stress. It is worth noting that with the occurrence of endplate failure conditions, the stress on the remaining endplate will increase. However, the maximum increasing trend of endplate stress with a long cage (only 0.17 Mpa) is significantly less than that with a short cage (the maximum is 1.5 Mpa).

The stress distribution on the cancellous bone of the upper surface of L5 is presented in Fig. 8B. Model 1 and 2 with Long cage showed that the highest stress did not exceed 0.34Mpa, and the lowest stress was only 0.14Mpa in Model 1 during left rotation. The maximum difference between Model 1 and 2 was 0.12Mpa. On the other hand, the model with short cage exhibited a significant increase in the stress of cancellous bone on the upper surface of L5 (up to 1.48Mpa), which was more prominent when the endplate was destroyed. Mode 3 had a maximum stress increase of 0.32Mpa and 0.27Mpa compared to Model 1 and 2, respectively, whereas the maximum stress of Mode 4 increased by 1.24Mpa and 1.17Mpa compared to Mode 1 and 2, respectively. The overall stress of cancellous bone on the upper surface of L5 of Model 1 and 2 was much lower than that of Mode 3 and 4. Furthermore, even with endplate damage, the stress borne by Model 2 was still lower than that of the model using short cage without endplate damage.

**Fig. 9:**
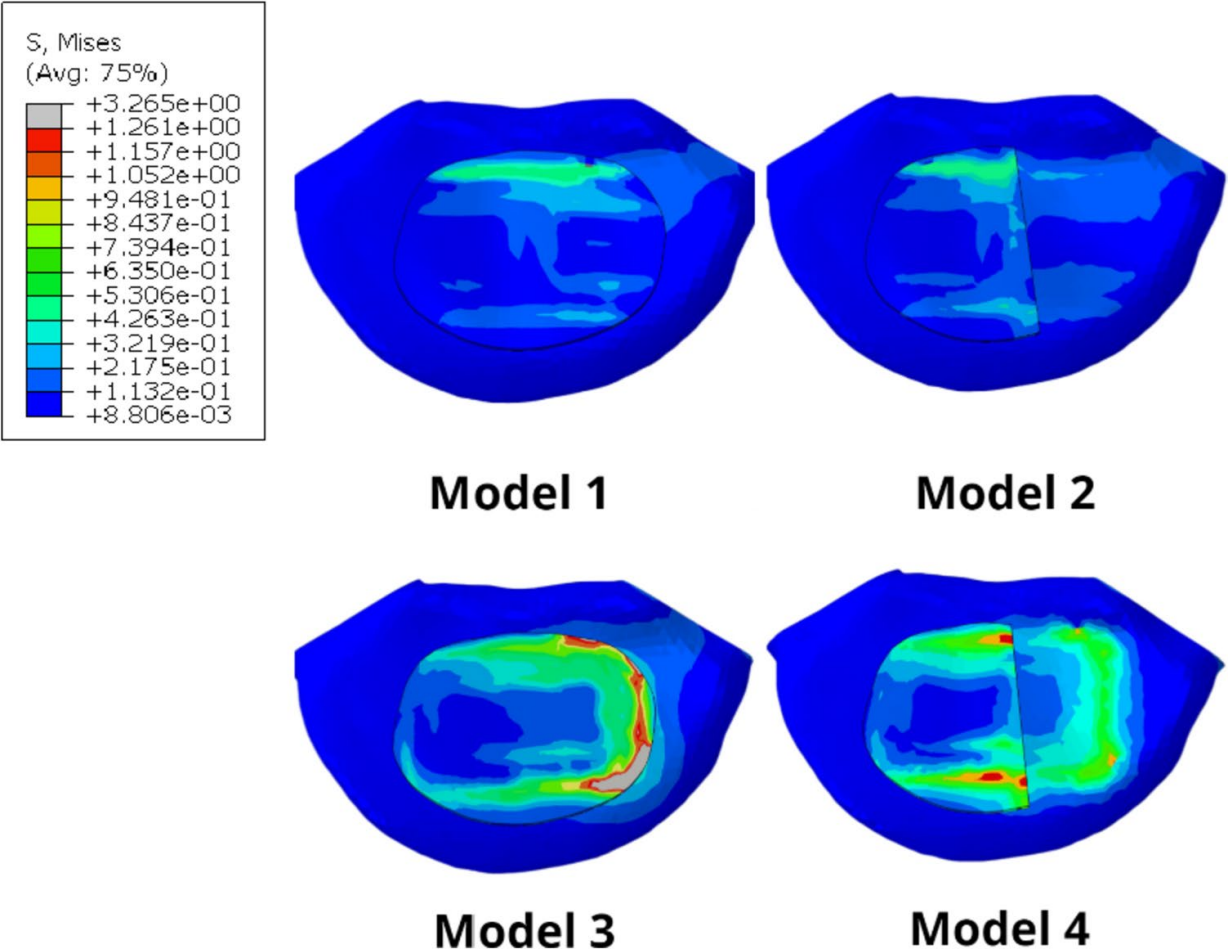
Comprehensive stress map of endplate and cancellous bone on the upper surface of L5

Figure 9 shows the integrated stress map of the L5 upper endplate and the cancellous bone on the L5 upper surface. The surface stress of Model 1 and 2 exhibits little difference in the figure and is significantly lower than that of Model 3 and 4. The maximum stress of the left endplate of Model 3 reached 3.3Mpa, which was significantly higher than that of the left upper surface of Model 4 (1.1Mpa).

## Discussion

Our study primarily focused on simulating lumbar lateral fusion surgery using finite element analysis. We compared the biomechanical properties between a long cage spanning the ring apophysis and a short cage covering only above the endplate. Our findings indicate that a lateral cage spanning the annular process can enhance segmental stability, improve compressive strength, and limit subsidence. These improvements have the potential to positively impact fusion and revision rates.

As patients age, an increasing number of them undergo lumbar interbody fusion to treat degenerative lumbar diseases, such as lumbar spinal stenosis, spondylolisthesis, degenerative disc disease, and adult spinal deformity [39, 40]. Lateral lumbar interbody fusion (LLIF), which includes extreme lateral interbody fusion (XLIF) and oblique lateral interbody fusion (OLIF), has become widely accepted due to its advantages such as less blood loss and faster return to work, compared to traditional posterior procedures such as posterior lumbar interbody fusion (PLIF) and transforaminal lumbar interbody fusion (TLIF) [41, 42].

The degree of fusion during the fixation stage has a definite effect on the postoperative outcome, but it cannot be analyzed from the perspective of imaging due to the nature of finite element analysis. However, according to the FDA definition of successful interbody fusion, successful interbody fusion is indicated by translational motion of the fusion segment less than 3mm, and ROM less than 5 [43]. The range of motion at the fusion level for all the models in this study was significantly less than 5°, indicating good immediate postoperative fixation. However, it was observed that the long cage significantly limits the range of motion of the fusion segment compared to the short cage. Additionally, the range of motion of the model using the short cage increased by approximately 5% in the presence of endplate destruction. Although the increase was not substantial, it reflects a postoperative trend. Therefore, the use of a long cage for fusion can reduce concerns regarding postoperative fusion.

A rigid fixation of the motion segment can cause loss of normal motion of the segment, resulting in compensatory increase in the range of motion of the adjacent segment and pressure in the intervertebral disc. This can accelerate degeneration and increase the risk of adjacent segment disease [44, 45]. Some previous studies have shown that an increase in range of motion (ROM) and intradiscal pressure (IDP) in adjacent segments can lead to the development of adjacent segment disease (ASD), and an increase in ROM in adjacent segments can increase the stress on adjacent intervertebral discs and the risk of adjacent segment degeneration. This is consistent with a study by Lee et al. [46], which demonstrated that LLIF can be beneficial in preventing the development of ASD through long-term follow-up of patients after the procedure, potentially due to its greater ability to restore postoperative sagittal balance. Moreover, anterior lumbar interbody fusion may aggravate ASD, and posterior lumbar interbody fusion is more unfavorable than anterior lumbar interbody fusion to aggravate ASD [47, 48]. This means that the two cages used in this study can reduce the risk of ASD in the adjacent segment during LLIF surgery.

Previous studies have indicated that the application of an interbody fusion cage and screw-rod system can establish an effective stress transmission pathway, allowing for stress distribution within the internal fixation system. The use of an interbody fusion cage can bear more pressure in the anterior column and reduce the stress on the screw-rod system, which was also confirmed by our study results. In our study, the stress on the screw-rod system exhibited a decreasing trend as the axial area of the cage increased in the postoperative model, and this trend was further amplified with the appearance of endplate destruction. The cage spanning the lumbar annular process had a greater bearing effect on the anterior column, which could disperse the pressure of the internal fixation system. However, as mentioned above, during the complex movements in daily life, the stress on the internal fixation system may increase, leading to the risk of internal fixation failure and screw-rod fracture. Nevertheless, the stress trend of the postoperative model screw-rod system also provides us with certain insights.

**Fig. 10:**
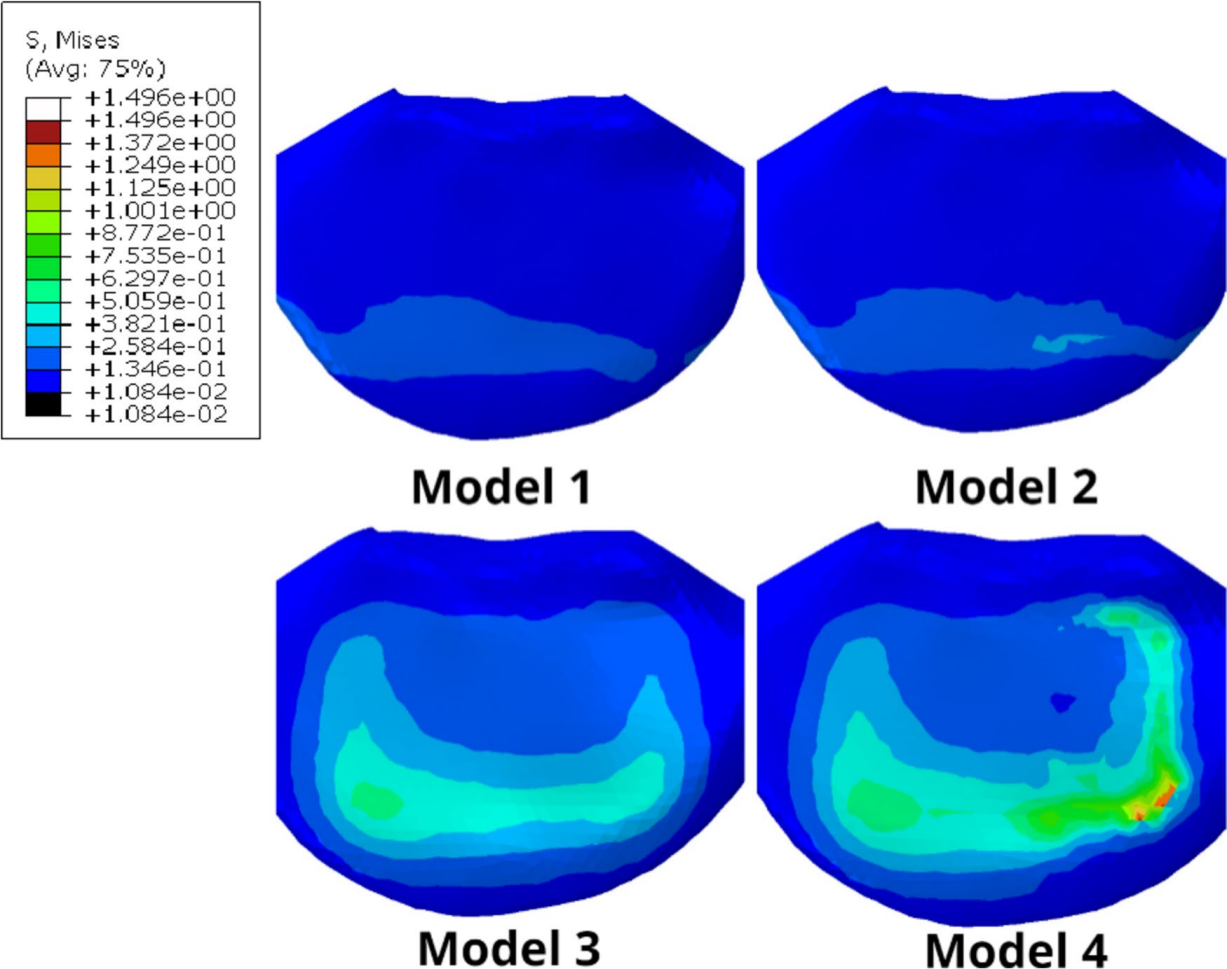
Cancellous bone stress on the upper surface of L5 during forward flexion

The importance of the endplate has been confirmed in many reports. Resection of the endplate can significantly reduce the structural properties of the lumbar vertebral body [8, 9]. Among them, endplate injury often occurs during endplate preparation and cage placement. Tatsumi Et al. [49] compared the endplate injury during cage placement through different lumbar fusion approaches. Although the possibility of endplate injury in the LLIF group was the lowest (4% of specimens), once it occurs, it can lead to cage subsidence, resulting in segmental lordosis and loss of foraminal height [50]. Oxland et al. [51] performed Indentation tests to verify the load-bearing role of the lumbar endplate. The results showed that the surface failure load and stiffness of the intact endplate were significantly different from those of the incomplete endplate (*P* < 0.0001). The average failure load after endplate removal was significantly reduced to 33% of the failure load of the intact endplate (*P* = 0.04), and the stiffness of the upper surface of the vertebral body was also significantly reduced (*P* = 0.01).In the study by Oxland et al.,, we found that the contact stress between the endplate and cage is the main factor causing implant subsidence. To compare the stresses at the interface between two different cages and the endplate, we simulated artificial endplate damage. The results showed that compared to Model 1, the endplate stress of Model 2 increased by a maximum of 0.17 MPa, while the endplate stress of Model 4 increased by a minimum of 0.4 MPa and a maximum of 1.9 MPa compared to Model 3. This indicates that as the supporting surface area of the endplate decreases, the remaining endplate bears more force, which may lead to more complications in incomplete endplates, consistent with previous studies. It should be noted that the endplate stress of Model 3 and 4 using a short cage is significantly higher than that of Model 1 and 2, regardless of the direction of motion, with a maximum difference of 6.9Mpa observed during the same maneuver. After the destruction of the endplate, the cage comes into direct contact with the vertebral cancellous bone. Therefore, we also evaluated the stress on the superior surface of the L5 cancellous bone. Without considering the right-tilting movement (where the stress is mainly concentrated on the right endplate, which was not removed in this study), the stress on the remaining incomplete endplates' superior surface cancellous bone significantly increased, with the most significant increase observed during flexion movement (Fig. 10). However, the overall cancellous bone stress was lower in models that used a long cage compared to those using a short cage, and even when the endplate was damaged, the stress increase was much smaller in the former than in the latter, as observed from the trend of stress increase from Model 3 to Model 4.

This suggests that the long cage allows for better force dispersion by spanning the vertebral ring apophysis, thereby reducing stress on the endplate and upper subchondral bone surface. Although the stress on the endplate in the postoperative model is much smaller than the failure strength of the cortical bone (90–200 Mpa) [33], and although our analysis is based on a specific condition, with real human activity being more complex, the results reflect the trend that this type of cage spanning the lumbar interbody process has a better anti-subsidence ability post-surgery.

Our study has certain limitations. Firstly, the data in this study were derived from the spine model of a 28-year-old adult male, and no statistical analysis was performed, which may lead to individual differences. This is a common limitation in finite element analysis. We have made slight simplifications in the model materials and assumed isotropic material properties for all structures. Additionally, the cage has been simplified without detailed consideration of its surface morphology. Consequently, these results may not fully reflect the biomechanical performance of the cage in different patients. In future research, we will pay more attention to the characteristics of materials. Moreover, we did not simulate the complex changes brought by muscles, which could not more accurately reflect the physiological characteristics of the normal lumbar spine. Secondly, it is regrettable that the data in this study are based on the normal bone population, and the osteoporosis population is not considered. Thirdly, we acknowledge the limitation of our study in not analyzing the stress and deformation of the facet joints. We recognize the importance of investigating this aspect and will address it specifically in future studies, focusing on standalone models. We plan to conduct more reasonable and rigorous biomechanical studies to verify our results.

## Conclusion

The endplate is a crucial structure in lumbar fusion surgery, and it is crucial to exercise special care during both endplate preparation and cage placement to prevent endplate damage. Our research on lateral lumbar interbody fusion procedures using a transvertebral ring apophysis cage(over-sizing the length of the cage by a minimum of 5mm wider than the radiographic pedicles) has demonstrated that this approach can effectively reduce stress on the internal fixation system and endplates, regardless of endplate integrity. Furthermore, this cage does not increase adjacent segment motion or intervertebral disc pressure. In contrast, cages placed only above the endplate can increase stress on the entire fixation system, leading to complications.

## Data Availability

Please contact the corresponding author for data requests.

## Abbreviations

FE: Finite element
ROM: Ranges of motion
IDP: Intradiscal pressure
ASD: Adjacent segment disease
PLIF: Posterior lumbar interbody fusion
LLIF: Lateral lumbar interbody fusion
TLIF: Transforminal lumbar interbody fusion
